# A world key to the genera of Elcanidae (Insecta, Orthoptera), with a Jurassic new genus and species of Archelcaninae from China

**DOI:** 10.3897/zookeys.954.52088

**Published:** 2020-07-29

**Authors:** Jun-Jie Gu, He Tian, Junyou Wang, Wenzhe Zhang, Dong Ren, Yanli Yue

**Affiliations:** 1 College of Agronomy, Sichuan Agricultural University, Chengdu, Sichuan, 611130, China Sichuan Agricultural University, Chengdu China; 2 College of Life Sciences, Capital Normal University, 105 Xisanhuanbeilu, Haidian District, Beijing, 100048, China Capital Normal University Beijing China; 3 Institute of Ecological Agriculture, College of Agronomy, Sichuan Agricultural University, Chengdu, Sichuan, 611130, China Inner Mongolia Museum of Natural History Hohhot China; 4 Inner Mongolia Museum of Natural History, No.13, South 2nd Ring Road, Saihan District, Hohhot City, Inner Mongolia, 010010, China Sichuan Agricultural University Chengdu China

**Keywords:** Jiulongshan Formation, Middle Jurassic, ovipostion, *
Sinoelcana
*, wing venation, Yanliao biota

## Abstract

A new fossil genus and species is described from the Middle Jurassic of China. The type of *Sinoelcana minuta***gen. et sp. nov.** has body and legs preserved. It is distinguished from all other elcanids by the unique combination of wing venation and stout ovipositor. The sickle-shaped ovipositor suggests that the new species had a preference for oviposition on plant material. A world key to the genera of Elcanidae is provided based on the wing venation.

## Introduction

Elcanidae Handlirsch, 1906 is the most diverse family of the enigmatic group Elcanidea. In the history of taxonomic study of elcanids, over a hundred species names were proposed, mostly based on the structure of their wings (Germar 1842; Giebel 1856; Handlirsch 1906–1908, 1939). After a critical investigation of wing venation, 104 species names in Elcanidae were considered to be invalid and were discarded from use (Zessin 1987). To date, Elcanidae consists of two subfamilies: Elcaninae Handlirsch, 1906 and Archelcaninae Gorochov, Jarzembowski & Coram, 2006 (Gorochov et al. 2006). Elcaninae, which are characterized by presence of a distal fusion among CuPaß, CuPb, and 1A, contains the genera *Probaisselcana* Gorochov, 1989; *Panorpidium* Westwood, 1854; *Eubaisselcana* Gorochov, 1986; *Cratoelcana* Martins-Neto, 1991; and *Minelcana* Gorochov, Jarzembowski & Coram, 2006. Archelcaninae are characterized by free distal part of CuPaß, CuPb, and 1A, and contains the genera *Parelcana* Handlirsch, 1906; *Synelcana* Zessin, 1988; *Archelcana* Sharov, 1968; *Sibelcana* Gorochov, 1990; *Hispanelcana* Penalver & Grimaldi, 2010; *Cascadelcana* Fang, Muscente, Heads, Wang, and Xiao 2018; and *Jeholelcana* Fang, Heads, Wang, Zhang, & Wang, 2018.

Northeastern China is rich and diverse in fossil insects (Zhang et al. 2010; Gu et al. 2012; Wang et al. 2012; Ren 2019). More than 60 species of Orthoptera have been reported from Yanliao and Jehol biota; however, only four are elcanids (Fang et al. 2015, 2018; Tian et al. 2019a, 2019b). Nevertheless, based on the compression fossil and amber collections of Elcanidae, this group exhibits a potentially higher diversity than expected (pers. obs.). Here, we describe a new genus with a new species of Elcanidae collected from Daohugou, Ningcheng, Inner Mongolia of China. This new finding enriches the diversity of Elcanidae and increases our knowledge of the wing morphology and reproduction behavior of this group. Furthermore, a world key to genera of Elcanidae, including this new genus and species, is provided based on wing venation characters.

## Method and materials

The specimens were examined with a Nikon SMZ 25 microscope and photographed with a Nikon DS-Ri 2 digital camera system. Line drawings were prepared using Adobe Illustrator CC 17.0.0 and Adobe Photoshop CC 14.0 graphics software. The measurements were taken using Adobe Illustrator. The specimens are housed at the Inner Mongolia Museum of Natural History, Hohhot, China.

Wing-venation analyses follow the interpretation proposed by Béthoux and Nel (2002). Notably, another venation system is also used to interpret the wing of Orthoptera (Sharov 1968; Gorochov 1995). The main difference is the interpretation between media and cubitus area. To make the wing constructions clear and unambiguous for readers, we list the other venation system used for Orthopera in brackets. Corresponding abbreviations used in taxonomical descriptions are as follows: CP (not covered), posterior costa; ScA (C), anterior subcosta; ScP (Sc), posterior subcosta; RA (RA), RP (Rs), anterior and posterior radius, respectively; MA (MA1), MP (MA2), anterior, posterior media, respectively; CuA (MP), CuP, anterior, posterior cubitus, respectively; CuPaα (CuA1), the anterior branch of first posterior cubitus; CuPaβ (CuA2), the posterior branch of first posterior cubitus; CuPb (CuP), the second posterior cubitus; AA1 (1A), first branch of anterior anal vein.

## Systematic palaeontology

### Class Insecta Linnaeus, 1758

#### Order Orthoptera Olivier, 1789

**Superfamily Elcanoidea Handlirsch, 1906**

**Family Elcanidae Handlirsch, 1906**

**Subfamily Archelcaninae Gorochov, Jarzembowski & Coram, 2006**

##### Sinoelcana

Taxon classificationAnimaliaOrthopteraElcanidae

Gu, Tian, Wang & Yue gen. nov.

45607787-6FFF-555A-8169-BBAAB591D8F6

http://zoobank.org/9E558599-9AFF-4FB6-A3A5-55E5E253893E

###### Type species.

*Sinoelcana minuta* Gu, Tian, Wang & Yue, sp. nov.

###### Etymology.

The generic name is a combination of the Greek prefix “sin-” (China) and *Elcana*. Gender: feminine.

###### Diagnosis.

Sickle-shaped ovipositor; meta-tibiae has leaf-like spurs; presence of two longitudinal veins between stem of RP and CuA+CuPaα; free CuPaα short, fused with M+CuA immediately after diverging from CuPa; CuPaα fused with M+CuA for a long distance.

###### Comments.

Based on the forewing venation, *Sinoelcana* gen. nov. can be assigned to Archelcaninae owing to its free distal parts of CuPaβ, CuPb, and AA1. The new genus is similar to *Sibelcana* Gorochov, 1990 and *Synelcana* Zessin 1988 by presence of two longitudinal veins between CuA+CuPaα and stem of RP, but it differs from *Sibelcana* in having a very short, free CuPaα and having CuA+CuPaα reaching the posterior wing margin, far beyond of the end of ScP; it differs from *Synelcana* in having a short, free CuPa, M, CuA, and CuPaα fused for a long distance, and narrow anals. *Parelcana* Handlirsch 1906 and *Cascadelcana* Fang, Muscente, Heads, Wang & Xiao, 2018 have the free CuA fused with CuPaα, which is much different from the new genus. Furthermore, the less numerous and spaced branches of the subcosta and radius, short CP, and more basal end of CuA+ CuPaα of *Cascadelcana* are quite different from the new genus. *Sinoelcana* differs from *Archelcana* Sharov, 1968 in that the latter only has one longitudinal vein between CuA+CuPaα and stem of RP. The type of *Sinoelcana* has leaf-like subapical spurs of meta-tibiae; the first three pairs are rather large. This kind of spurs is also present in another Chinese elcanid genus *Jeholelcana* Fang, Heads, Wang, Zhang & Wang, 2018, but differs from *Hispanelcana* Penalver & Grimaldi, 2010. *Sinoelcana* can be distinguished from *Jeholelcana* by its three branches of M and short CuPa.

##### Sinoelcana minuta

Taxon classificationAnimaliaOrthopteraElcanidae

Gu, Tian, Wang & Yue sp. nov.

CDEB8278-22F6-5E41-8657-D4B53959583B

http://zoobank.org/874D154C-777D-4254-8972-7622A13A2413

###### Diagnosis.

As for genus.

###### Materials.

***Holotype***: IMMNH-PI11334 (Part), IMMNH-PI11335 (Counterpart), Female.

###### Locality and age.

Daohugou Village, Wuhua Township, Ningcheng County, Inner Mongolia, China; Jiulongshan Formation, Bathonian–Callovian boundary interval (Xu et al. 2016; Yang et al. 2020), Middle Jurassic.

###### Description.

**Figure 1.**
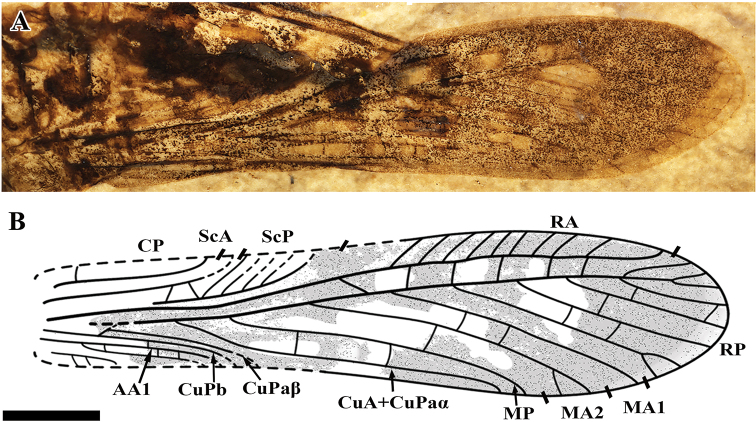
Holotype of *Sinoelcana minuta* sp. nov., IMMNH-PI11334, forewing. **A** Photograph **B** reconstruction drawing. Scale bar: 2 mm.

**Figure 2.**
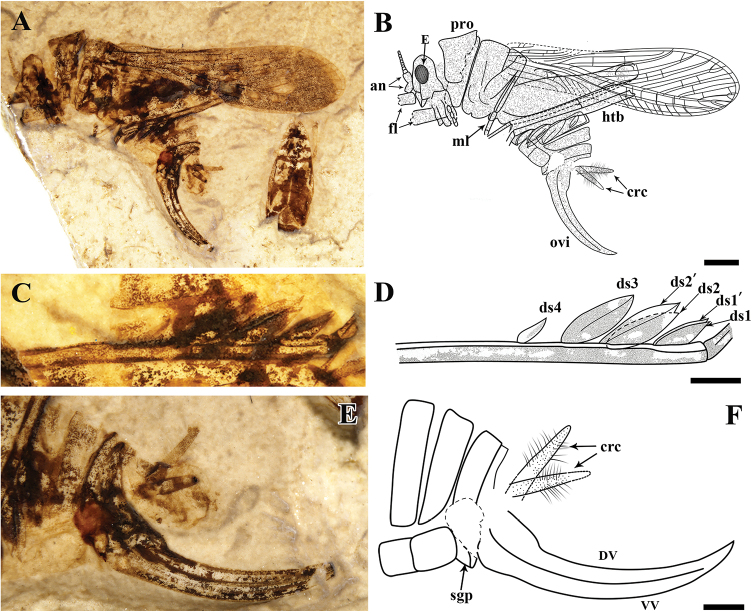
Holotype of *Sinoelcana minuta* sp. nov., IMMNH-PI11334. **A, B** Habitus, photograph (**A**) and reconstruction drawing (**B**). **C, D** meta-tibiae, photograph (**C**) and reconstruction drawing (**D**). **E, F** ovipositor, photograph (**E**) and reconstruction drawing (**F**). Scale bars: 2 mm (**A, B**); 1 mm (**C–F**).

Head: head hypognathous, with large, oval eyes; scape cylindrical, much wider than pedicel and the flagellum; compound eyes rather large, 1.1 mm long, oval; Thorax (Fig. 2A, B): pronotum saddle-shaped, 2.4 mm long, lateral lobe 2.6 mm high. Legs: meta-femur 8.1 mm long, 1.9 mm wide; meta-tibiae has three pairs of large, leaf-like spurs, and one basal and small spur, ds3 1.86 mm long, ds2 1.86 mm long, ds1 1.25 mm, ds4 0.85 mm long (Fig. 2C, D). Forewing (Fig. 1): 14.3 mm long, 3.4 mm wide (maximum width recorded); CP distally curved and reaching anterior wing margin beyond the origin of CuA+CuPaα; ScA simple, ending in anterior margin nearly 1/4 of the wing length; ScP reaching anterior margin basal of the origin of stem RP and giving off 5 long and oblique branches ending in anterior margin; stem R long and strong, branched into RA and RP close to the middle of wing length; area between ScP and R basally narrow, getting wider after ScP reaching wing margin; RA has numerous oblique branches reaching anterior margin; RP fused with MA1 distal to the end of ScP; RP has 6 main pectinate branches and 8 terminals; M forking into MA and MP at the level of the end of ScA; MA forking into MA1 and MA2 at the level of the end of ScP; the fusion of RP and MA1 distant to the origin of MA1, MA2 distally branch; MP simple, originates at the level of the end of ScP; area between branches of RA and RP covered with simple and straight crossveins; CuA+CuPaα simple, slightly undulate, originating basal of the end of CP; CuPa short, forking into CuPaα and CuPaβ close to the wing base; CuPaα fused with M+CuA immediately and running for a long distance; CuPaβ and CuPb simple; AA1 strong, reaching posterior wing margin distal of the end of ScA; without distinct coloration spots on the wing. Abdomen: cerci short and conical, unsegmented, 2.3 mm long, with numerous hairs; ovipositor sickle like, 6.4 mm long (measured from the tip to the base), more than twice as long as the pronotum.

## Discussion

The ovipositors of ensiferans are always specially modified related to the site and pattern of oviposition (Gwynne 2001; Rentz 2010). Currently, only a few fossil elcanid species have ovipositors preserved, which exhibit a straight, elongated, and sharply pointed shape (Zessin 1987; Martins-Neto 1991; Tian et al. 2019a). Although the ovipositors of these insects are quite different in their measurements, their shapes are similar and sword-like. These similarities to extant Ensifera imply that laying eggs in the ground/soil was a common behavior of Mesozoic elcanids. In contrast with the ovipositor structural design above, *S. minuta* Gu, Tian, Wang & Yue sp. nov. has a comparatively short and stout ovipositor. The ovipositor is slightly curved and its apical portion of dorsal valvulae is smooth and without any serrations (Fig. 2E, F). This kind of sickle-shaped ovipositor indicates that the species oviposits on plant material, either dead wood or stems (Rentz 2010).

Currently, 11 genera are attributed to Elcaninae and Archelcaninae (Gorochov et al. 2006). Among them, *Cratoelcana* were described with two new species including females and males from Crato, Brazil. These specimens are exquisite and almost completely preserved, but with greatly overlapped wings. Based on the reconstructions of the wing, it is worth examining the area of the cubitus and anal veins, which is not common in general for fossil elcanid species. From the drawing of specimen CV-1098 of *Cratoelcana zessin* (Martins-Neto 1991), the reconstruction brings a unique fusion with CuPb and AA1, but the interpretation might need a more thorough examination of the specimen. Hence, the present subfamily assignment should be regarded with caution.

*Jeholelcana yixianensis* was described from the Jehol biota and presented with a very unique character: specifically a long and oblique free CuA vein fused to the CuPa vein, which was treated as a diagnostic character for the species and genus (Fang et al. 2018). This condition is very peculiar in Elcanidae and even among orthoperans in general. Fang et al. (2018) followed the nomenclature proposed by Béthoux and Nel (2002); however, they made an incorrect interpretation regarding the venation. Based on the reconstruction of the wing, the cubitus part exhibits a typical pattern of Elcanidae where CuPa basally forks into CuPaα and CuPaβ, and then CuPaα fuses with M+CuA. In other words, the vein CuA+CuPa interpreted by Fang et al. should be CuA+CuPaα. Furthermore, the forewing shows an unusual condition in that CuPaβ fuses with CuA+CuPaα for short distance. It is not common in orthoperans, if we treat it as a stable character state, but some similar conditions were documented in several relatives of orthoperan species. *Longzhua loculata* exhibits an unusual condition in which a branch of CuA fuses with the posterior branch of M (Gu et al. 2011). Based on more than 60 samples of forewings, and with only two specimens have a branch of CuA fusing to the posterior branch of M, and this condition is reasonable to interpret as a translocation of a vein or a consequence of fusion, rather than a unique character state (Gu et al. 2011). The same situation occurs in another Carboniferous archaeorthopteran species, *Miamia maimai* (Béthoux et al. 2012). Regardless, for extant orthopterans, this condition is also present among winged caeliferans and ensiferans. To verify this assumption, we examined six wing pairs of *Calliptamus abbreviates* and found that one of them exhibited a CuPaβ distally fused with CuA+CuPaα, whereas the CuPaβ of the remainder of the specimens examined were distant to CuPaα (unpublished data). Therefore, CuPaβ fused with CuA+CuPaα is not a suitable diagnostic character for *Jeholelcana*.

Due to the rare occurrence of complete wingsets of Elcanidae and the typical requirements for a large sample of species to establish wing venation characters, taxonomy and further phylogenetic work in the Elcanidae are challenging. As more new materials are discovered, a comprehensive rechecking of the classification of known species worldwide is needed. Presently, there are six amber-embedded species attributed to Elcanidae (Poinar et al. 2007; Peñalver and Grimaldi 2010; Heads et al. 2018). Lack of wing preservation has made establishment of their subfamily positions hard to confirm. From the current database of Orthotpera species (Cigliano et al. 2020), *Elcanopsis sydneiensis* Tillyard, 1918 and *Macrelcana ungeri* (Heer, 1849) are presently included within the Elcanidae. However, *Elcanopsis sydneiensis* is only known for a fragment, which is probably the distal part of the forewing of an elcanid-like insect (Tillyard 1918). *Macrelcana ungeri* (Heer, 1849) is lacking the diagnostic characters of Elcanidae based on the reconstruction of the wing (Karny 1932). In conclusion, we propose a key to the genera of Elcanidae based on forewing venation characters where the amber-embedded taxa and *Cratoelcana* are not considered.

### Key to the genera of fossil Elcanidae based on wing venation

**Table:** 

1	Area between RA and RP widened; CuPaβ, CuPb, and AA1 without fusion	**2**
–	Area between RA and RP not widened; CuPaβ, CuPb, and AA1 distally fused or just CuPaβ fused with CuPb	**8**
2	CuPaα fused with free CuA	**3**
–	CuPaα fused with M+CuA	**4**
3	Presence of three longitudinal veins between CuA+CuPaα and stem of RP	*** Parelcana ***
–	Presence of two longitudinal veins between CuA+CuPaα and stem of RP	*** Cascadelcana ***
4	M has two branches, forming MA and MP	*** Archelcana ***
–	M has more than two branches, MA branched	**5**
5	MA has three main branches	*** Jeholelcana ***
–	MA has two main branches	**6**
6	Free CuPa long, slightly arched to the anterior wing margin	*** Synelcana ***
–	Free CuPa short, directed towards the anterior wing margin	**7**
7	CuPa vertically diverges from CuP; CuPaα fused with M+CuA and separated from the fusion with CuA immediately	*** Sibelcana ***
–	CuPa obliquely diverges from CuP; CuPaα fused with M+CuA for a long distance	***Sinoelcana* gen. nov.**
8	CuPaβ, CuPb, and AA1 distally fused	**9**
–	CuPaβ distally fused with CuPb	*** Eubaisselcana ***
9	Area between MP and posterior wing margin broad and covered by oblique, regular, long cross-veins	*** Minelcana ***
–	Area between MP and posterior wing margin narrow, without long, oblique cross-veins	**10**
10	M with three branches	*** Probaisselcana ***
–	M with more than three branches	*** Panorpidium ***

## Supplementary Material

XML Treatment for Sinoelcana

XML Treatment for Sinoelcana minuta
